# Dental Erosion Evaluation with Intact-Tooth Smartphone Application: Preliminary Clinical Results from September 2019 to March 2022

**DOI:** 10.3390/s22145133

**Published:** 2022-07-08

**Authors:** Andrea Butera, Carolina Maiorani, Simone Gallo, Maurizio Pascadopoli, Sergio Buono, Andrea Scribante

**Affiliations:** 1 Unit of Dental Hygiene, Section of Dentistry, Department of Clinical, Surgical, Diagnostic and Pediatric Sciences, University of Pavia, 27100 Pavia, Italy; andrea.scribante@unipv.it; 2 Unit of Orthodontics and Pediatric Dentistry, Section of Dentistry, Department of Clinical, Surgical, Diagnostic and Pediatric Sciences, University of Pavia, 27100 Pavia, Italy; simone.gallo02@universitadipavia.it (S.G.); maurizio.pascadopoli01@universitadipavia.it (M.P.); 3 Founder and Technical Director of AddWare Europe Ltd., Thremhall Park, Start Hill, Bishop’s Stortford CM22 7WE, UK; sergiobuono@icloud.com

**Keywords:** BEWE index, caries, dental erosion, Intact-Tooth, learning machine

## Abstract

Dental erosion is a process of deterioration of the dental hard tissue; it is estimated that about 30% of permanent teeth are affected in adolescence. The Intact-Tooth application allows for the better estimation of the problem, inserting itself in the diagnosis process, and better care and prevention for the patient. It provides him with scientifically validated protocols, which the patient can consult at any time. The purpose of this report was to conduct an initial evaluation on the use of the application, which has been available since September 2019: the analysis of the collected data allowed the first investigation of the incidence of the problem and the degree of susceptibility in the registered patients. Photos of 3894 patients with dental erosion were uploaded, through which the degree of susceptibility and the BEWE (basic erosive wear examination index) index could be assessed; of these, 99.72% had a susceptibility grade of 0 to 8, while 0.28% had a medium-high susceptibility grade; this result is related to the age and sex of the patients. The management of patients through the help of the application could promote the diagnosis and treatment of enamel diseases and encourage the self-learning of the learning machine, thanks to the number of clinical cases uploaded.

## 1. Introduction

In recent decades there has been growing interest in dental erosion in clinical practice, public health and dental research, since its prevalence is still increasing, especially in the younger age group: it varies from 13–53% in the different populations examined; according to the AIC (Italian Academy of Conservative Dentistry), it affects 40% of Italians and, despite the high incidence, it is still underestimated [1]. In terms of overall prevalence, a systematic review estimated that 30% of permanent teeth in an age range of 8 to 19 years were affected by dental erosion [2]; furthermore, it would appear that hard tissue erosion increases with age and becomes more severe [3,4].

From the clinical point of view, it is a process with multifactorial aetiology, characterized by the progressive loss of dental hard tissue caused by food or acid substances without bacterial involvement. Incorrect habits, such as frequent alcohol intake, cigarette abuse and eating disorders, together with the acidic pH of some foods, weaken the enamel and consequently make the tooth structure more fragile. In addition to endogenous and exogenous causes, others can lead to abrasion of the tooth’s hard tissues: malocclusion, bruxism and incorrect use of the toothbrush during home oral hygiene [5,6].

If not properly treated, there are problems both from an aesthetic point of view, as the enamel surface appears translucent, and from a functional point of view, as the exposure to dentin causes dentinal hypersensitivity and increased risk of developing carious lesions. Treatment depends on the stage of the lesion and is simple in the early stages of enamel loss (basic erosive wear examination index, BEWE 3–8), when it is treated with minimally invasive direct restorations with composites and fluoridated enamel. In the middle stages, when the dentin is exposed (BEWE 9–13), treatment involves direct and indirect crown restorations, while, in the advanced stages, with more than 50% destruction of the tooth surface (BEWE 14–18), treatment consists only of indirect prosthetic reconstruction; in the advanced stages, pulpal chamber opening may be necessary, followed by endodontic treatment, exposing the tooth to an increased risk of fracture due to altered tooth metabolism and loss of vitality [7,8].

Today, a new support tool for managing the phenomenon of dental erosion is available: an app called Intact-Tooth; this application is public, developed by AddWare Europe Limited, Thremhall Park Start Hill, Bishop’s Stortford Hertfordshire, CM22 7WE, United Kingdom (https://apps.apple.com/it/app/intact-tooth/id1467364505; https://play.google.com/store/apps/details?id=com.addware.intact_tooth (accessed on 1 September 2019)).

Intact-Tooth is aimed both at oral health professionals, to whom it offers a clinical support tool, and at patients, to whom the personalized home oral hygiene indications issued by professionals are addressed, as well as at the world of research for the creation of a documented referral database for images. The Intact-Tooth App supports the professional in evaluating the progress of erosive processes, offering the possibility of defining the indicative degree of dental erosion and classifying the susceptibility of each subject. In addition, it allows the creation of of a patient archive, on which to record the reference indices and images to evaluate the presence of lesions on the enamel surface. Furthermore, there is a collection of therapeutic protocols, scientifically validated, oriented to remineralization and desensitization of the teeth, which operators are then free to modify and integrate. The platform has been conceived according to an artificial intelligence management model. To date, it is possible to evaluate the BEWE index (basic erosive wear examination index) and to calculate induced susceptibility thanks to a mode of graphically assisted interaction or numerical scoring. The one with numerical input substantiates the experience of the professional. Therefore, it will be possible to use it to “train” the expert system on the recognition of dental elements (segmentation), laying the foundations for the automatic assisted recognition of erosion zones. It should be noted that, in any case, the practitioner may revise the proposed result, and it will also be possible (considering the sub-set of fixed recognition) to improve the accuracy and reliability of the expert system in discriminating erosion zones on a photo.

Therefore, the goal of this application is to evaluate enamel integrity and the education of the learning machine by loading clinical data, and it is the purpose of this report to conduct an initial evaluation on the use of the application and to show the first data collected regarding its use by health professionals and patients; of the latter, data on the degree of susceptibility and the BEWE index are shown, indicating a preliminary analysis of the dental elements affected by erosion.

## 2. Materials and Methods

To conduct an initial evaluation of the application, all data in the database (The SQL database is MariaDB 10 (MariaDB Corporation Ab, Tekniikantie 12, 02150 Espoo, Finland) in the implementation for DMS [Synology]; https://www.synology.com/it-it/dsm/packages/MariaDB10?os_ver=7.1 (accessed on 9 June 2022)) in September 2019 were collected: number of health care users [dental hygienists (1231), dentists (463) and students (145)] and number of patients (3894). Additionally, information on several patients in orthodontic and non-orthodontic therapy, BEWE index (basic erosive wear examination index), and susceptibility were also collected [9,10].

In the care and management of the patient, at a professional level, it is possible and useful to perform a salivary screening that allows, together with reference indices such as Bewe (an index that evaluates the state of dental erosion) and Schiff air index (an index that assesses the state of dentinal sensitivity), to classify the patient in terms of risk:

- Mild Susceptibility: good salivary amount (>5 mL), with pH between 6.7 and 7.8, good buffering capacity, BEWE index between 0 and 1 and Schiff air index between 0 and 1.
- Moderate Susceptibility: small amount of saliva (including 5–3.5 mL), with pH between 6.0 and 6.6, good buffering capacity, BEWE index between 1 and 2 and Schiff air index between 1 and 2.
- Severe Susceptibility: There is little or no salivary amount (<3.5 mL), with pH < 6.6, and fair/poor buffering capacity, BEWE index between 2 and 3 and Schiff air index between 2 and 3.

The evaluation is done from one or more photos of the patient’s teeth.

**Figure 1:**
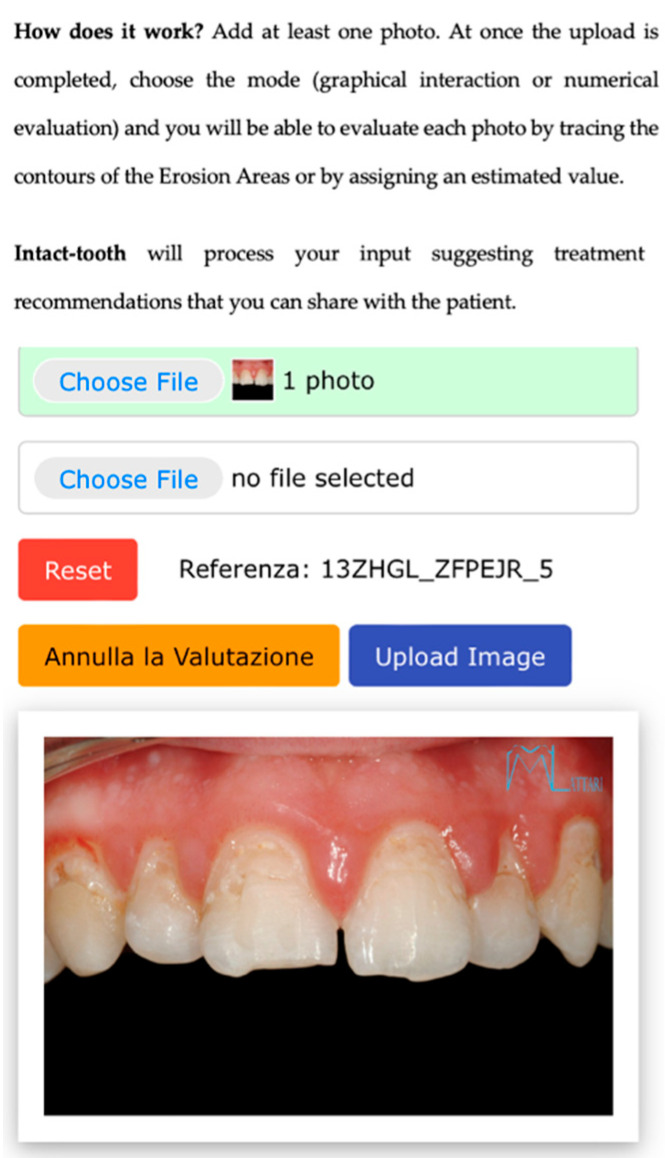
Shows the initial screen for evaluation, allowing the patient’s photo to be uploaded.

**Figure 2:**
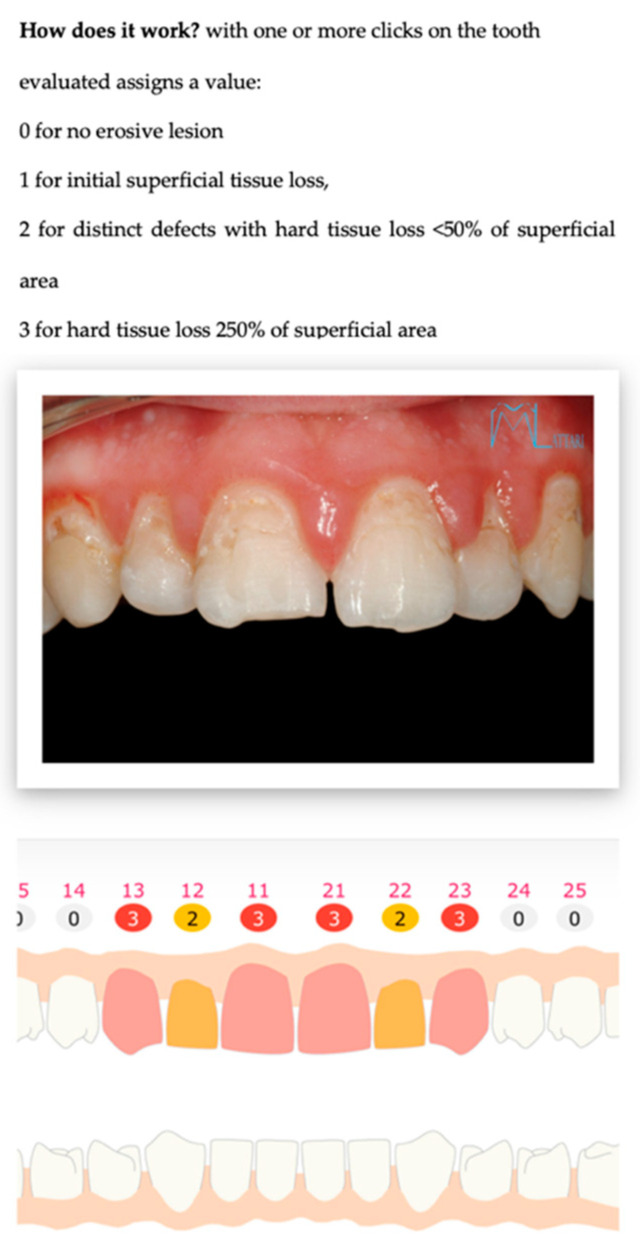
Shows the numerical assessment of erosion present on the elements, allowing a value from 0 to 3 to be awarded.

**Figure 3:**
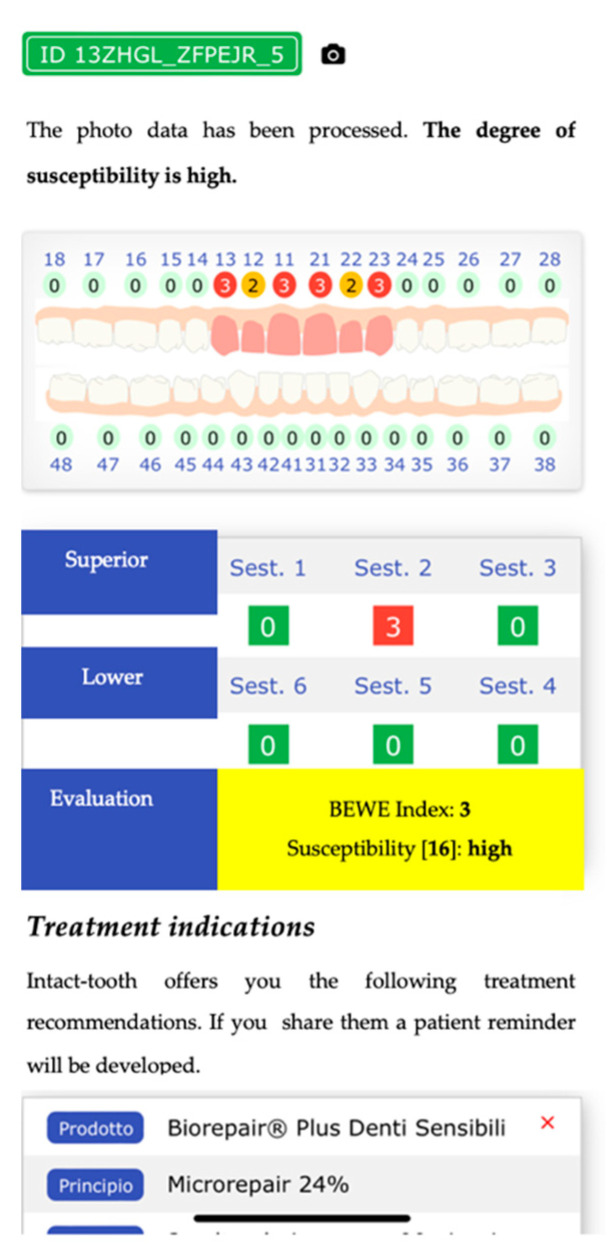
Shows an odontogram with the value of the erosion status, adjudicated by the healthcare professional, and provides the calculation of the BEWE index and the degree of susceptibility, with the most appropriate protocols to treat the patient over time.

For each photo, the evaluator can take two approaches:

- Numerical. By interacting on an odontogram, the practitioner can indicate the estimated erosion level for each element. This action populates a database, and then an evaluation algorithm calculates the BEWE and susceptibility indexes Figure 1, Figure 2 and Figure 3.

With one or more clicks on the affected elements, the practitioner associates an erosion value. On the one hand, this approach enables evaluation and populates a database of evaluated images representing a collection of reusable noumena to train the future artificial intelligence machine.

**Figure 4:**
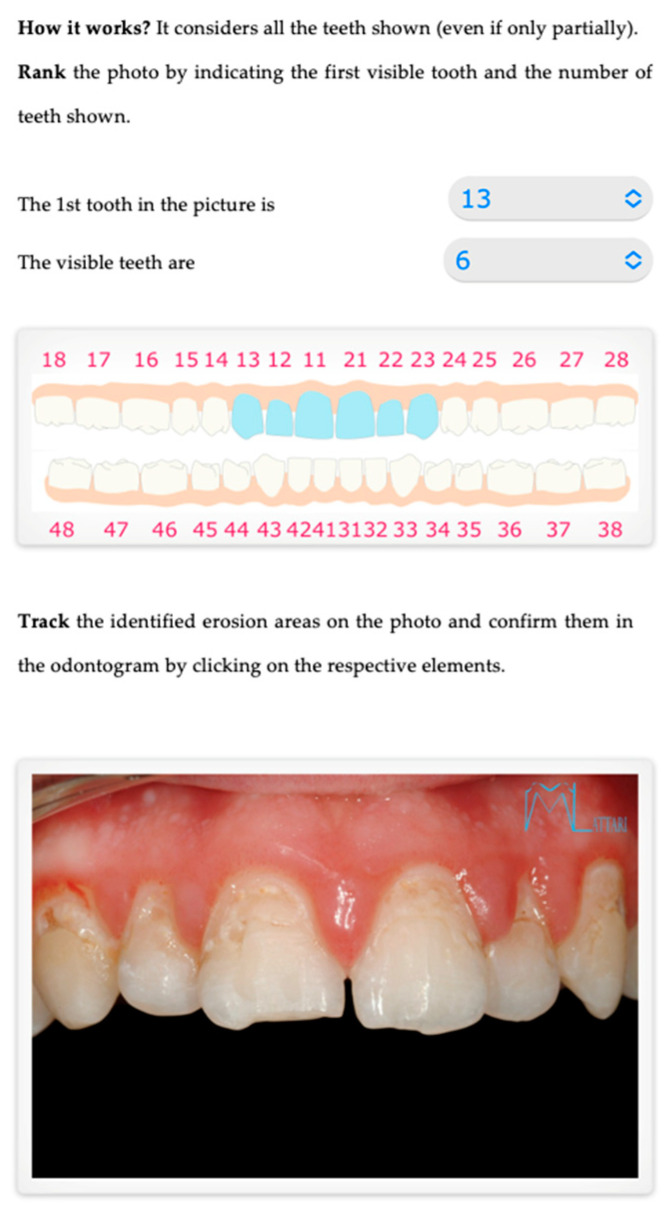
Shows the initial screen for evaluation, where you can indicate the dental elements visible in the patient’s photo uploaded to the system.

**Figure 5:**
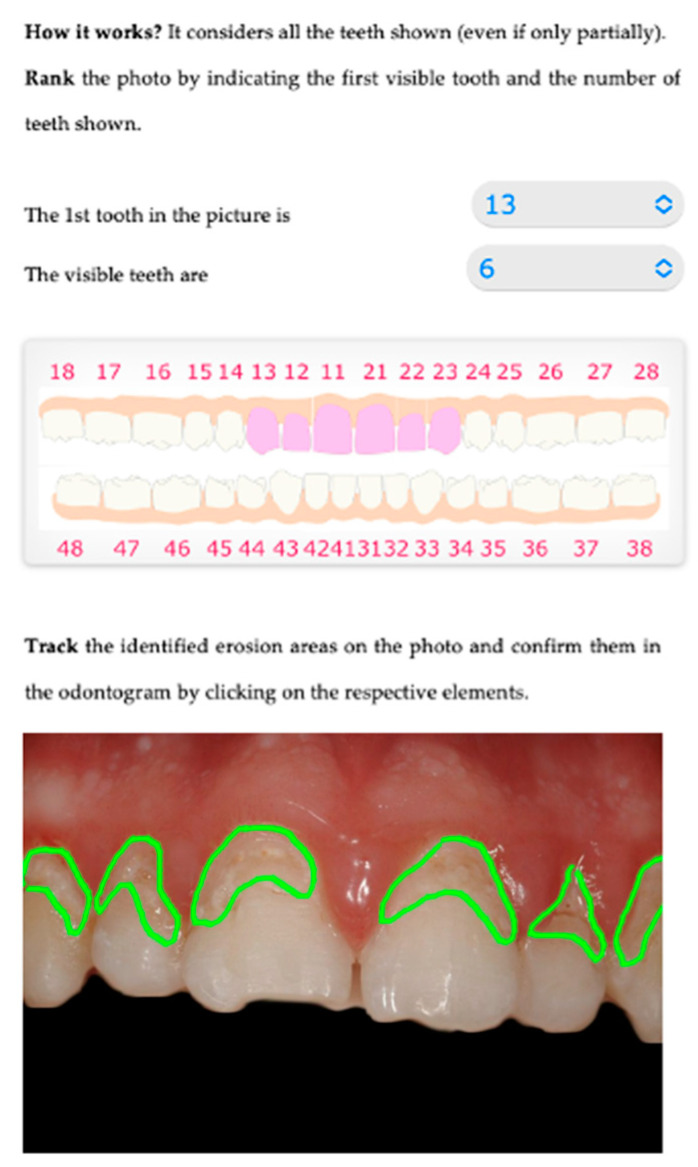
Shows the graphical/assisted assessment, indicating the number of elements present in the photo and subsequently tracing the areas of enamel affected by erosion.

**Figure 6:**
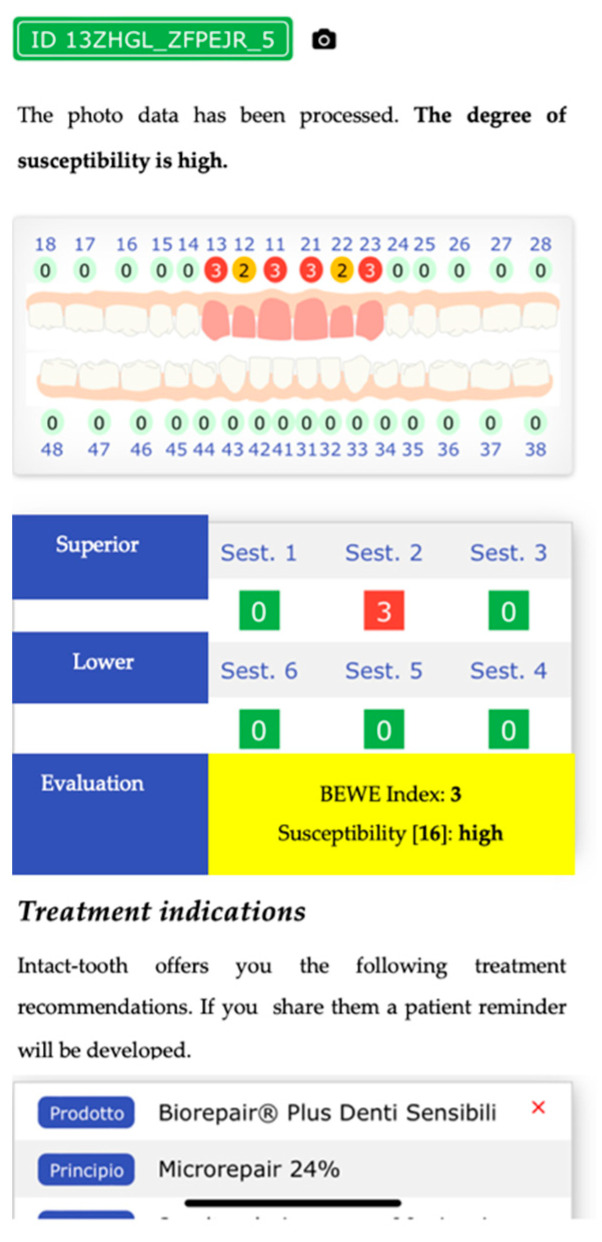
Finally, shows an odontogram with the value of the erosion status awarded by the system and provides the calculation of the BEWE index and the degree of susceptibility, with the most appropriate protocols to treat the patient in time.

- Graphical. The practitioner identifies on an odontogram the elements to be considered and draws the outline of the detected erosion area on the photo. This action provides Intact-Tooth with two files, the original photo and the drawing of the contour areas. Figure 4, Figure 5 and Figure 6.

With the understanding that the discernment of erosion is always left to the practitioner’s experience, this method involves memorizing several additional parameters compared to the previous numerical approach. These parameters will be helpful in future development.

For each photo, the practitioner indicates the first visible element and the number of teeth displayed. Then, he graphically outlines the detected erosion area for each detected element. Here, it is essential to specify that the practitioner draws on a transparent layer superimposed on the original Intact-Tooth image for each photo, acquires two images (the initial picture and the graphic drawing) and the notion of the numberings of the elements involved. The two images have the shape factor normalized to the exact width of 1472 px. A map is extracted from the baseline photo, detecting only the area related to the teeth; the map is then segmented into sub-areas for the dental elements (graphical processing is done using OpenCV (Open Source Vision Foundation. 445 Sherman Ave #150, Palo Alto, CA 94306, USA)). The procedure calculates the number of pixels contributing to each sub-area. For each sub-area, the number of pixels is counted, and an array of data is returned, the first value of which refers to the tooth classification number associated by the practitioner and the subsequent ones, sequentially.

Similarly, using the same method, the number of pixels concurring in each identified area is counted from the graphic layer on which the contours of the erosion areas were plotted. The procedure returns a data array whose first element is the classification number of the first tooth on which erosion was indicated and the subsequent ones in sequence. Comparison of the significant positions of the two arrays yields the percentage of erosion for the individual tooth with which a score is associated that is significant for the attribution of the BEWE value and associated susceptibility.

**Figure 7:**
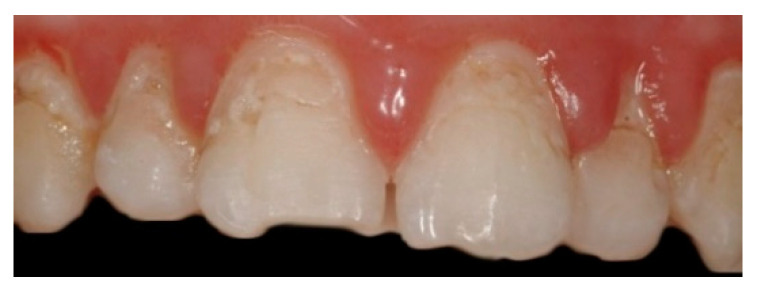
Is the original photo.

**Figure 8:**
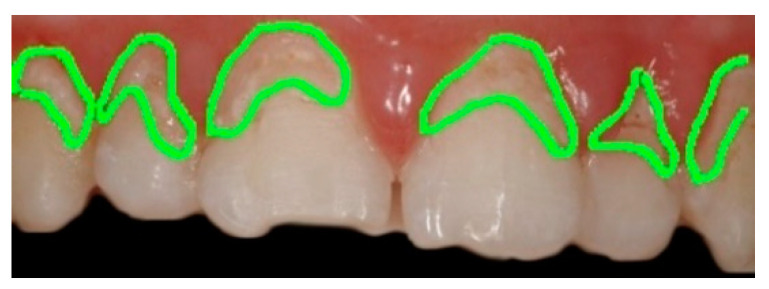
Shows the original image with the traced contours of the erosion area.

**Figure 9:**
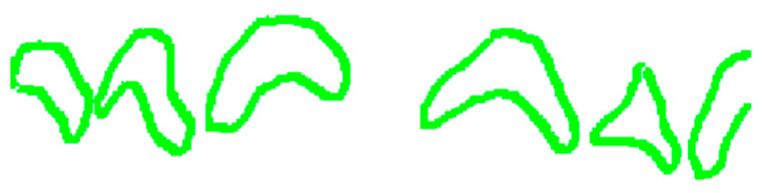
Shows the contours of the erosion area, extrapolated from the main image.

**Figure 10:**
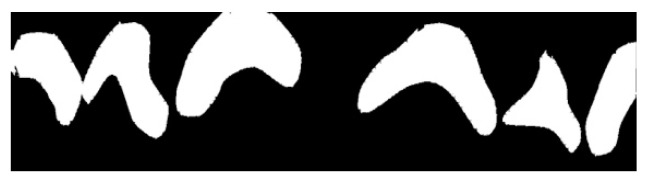
Shows the erosion area.

**Figure 11:**
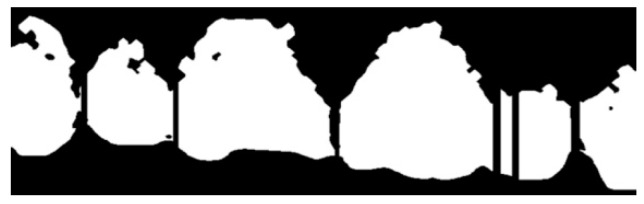
Shows the area of healthy tissue.

So, an image processing procedure performs these operations in sequence (Figure 7, Figure 8, Figure 9, Figure 10 and Figure 11):

- the two files are normalized in the represented graphic dimensions;
- the original photo is segmented, i.e., the individual elements of which it is composed are rendered treatable;
- for each segment of the original photo, the area in pixels is calculated;
- similarly, the drawing is segmented, and for each segment, the site is calculated.

**Figure 12:**
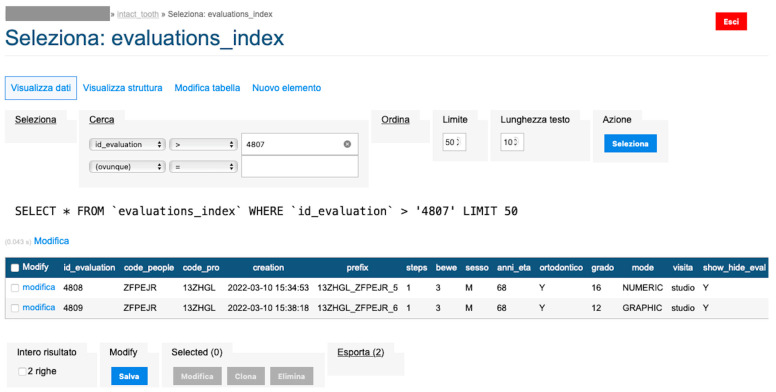
Shows the first part of evaluation index process.

**Figure 13:**
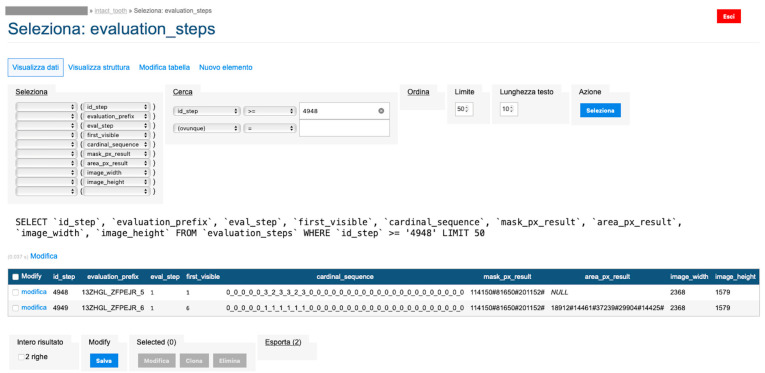
Shows the second part evaluation index process.

An algorithm evaluates the data for each element by calculating the score attributable to each tooth by the difference in area values. The data are stored and flow into the database mentioned in the previous numerical approach with the exact calculation of the BEWE index and susceptibility index (Figure 12 and Figure 13).

Sensitive patient data are encrypted with private keys at the level of each practitioner.

Figure 7, Figure 8, Figure 9, Figure 10 and Figure 11 show the segmented image, indicating the amount of healthy and ill tissue.

Consequently, regardless of the approach, a subsequent and final algorithm proposes a set of treatment indications that the practitioner can modify.

Once the indications are confirmed, Intact-Tooth conveys them to the patient along two lines:

- Sending a link to the evaluation from SMS to the patient’s smartphone;
- Sending a push notification to the patient’s application if the patient is a user of the competing application (MyOralCare.app, AddWare Europe Ltd., Thremhall Park, Star Hill Bishop’s Stortford CM22 7WE UK).

### Backend

The backend is based on an Apache webserver published on a high-availability cluster (SHA). Transactions with the Intact-Tooth database are done in SQL; the database is MariaDB10.

- The database of the concurrent application (MyOralCare) is PostgreSQL and contains a specific table alignment to the MariaDB10 data structure.
- The backend applications are developed in PHP and support multiple database connections.
- The user applications are hybrid (Ionic framework) and implement native functions through a set of plugins compatible with Capacitor.
- The treatment of the images is implemented on a dedicated machine to which the backend transfers the acquired files and from which it receives in numerical form the calculated values of the original area and contour area.
- The hardware involved is located in a TIER III data centre with ISO 27001, ISO 14001 and ISO 50,001 certification.

Computer software was used to calculate descriptive and inferential statistics (R version 3.1.3, R Development Core Team, R Foundation for Statistical Computing, Wien, Austria). Descriptive statistics were calculated. Linear regressions were performed to test the effect of different variables (sex, age and orthodontic treatment) on susceptibility and grade. Significance was predetermined at *p* < 0.05 for all tests.

## 3. Results

Descriptive statistics about software users and patients are reported in Table 1, Table 2, Table 3, Table 4 and Table 5.

Linear regression showed the presence of a significant effect of grade on susceptibility (*p* < 0.0001).

Sex (*p* = 0.0005) and age (*p* = 0.0019) showed a significant effect on grade, whereas orthodontic treatment showed no significant effect on grade (*p* > 0.05).

## 4. Discussion

The goal of the Intact-Tooth application is to investigate and analyze tooth hard tissue pathologies, which are often underestimated due to a lack of diagnosis.

The app has been used by a total of 1839 professionals (dentists, dental hygienists, dental students and dental hygiene students), with 3894 patients registered as of September 2019; results showed that age and sex have an effect on grade of susceptibility. These are the first data collected, confirming knowledge about the segment of the population most affected by dental erosion [1,2,3]. These results should be understood as a starting point for diagnostic work and as a starting point for evaluating the collected results of each individual patient, assessing any improvements in the future as a result of the treatment and maintenance protocols provided. In addition, for future reports on the application and to improve it, it would be useful to provide questionnaires to both clinicians and patients to facilitate the application’s simplicity, use, and satisfaction [11].

The future perspective, seeing the data achieved so far, is to provide a complete system, which will only be possible by continuing to add clinical cases, so that the application can differentiate eroded enamel from healthy enamel in full autonomy, thanks to the learning machine education that will be refined with each clinical case uploaded to the application. Beyond the immediate purpose of evaluating the photo, the system populates a database of estimated images that will allow the artificial intelligence machine to learn and apply the notion of recognition and classification of dental elements and to identify areas of suspected or apparent erosion, suggesting to the practitioner an interpretation of the photo based on accumulated experience. While Intact-Tooth is definable as an assisted diagnostic tool, in the future, when the AI machine is implemented and sufficiently trained, Intact-Tooth would make the collective prior clinical experience available to the practitioner and take on the value of an accurate, comprehensive diagnostic tool.

Unfortunately, after extensive research, there are no similar apps on IOS and Android; Intact-Tooth can interact with another free downloadable app, MyOralCare. This application is designed to disseminate valuable content to the praesidium of oral health through interactive awareness methods, exposing tips and insights and structured knowledge derived from dental hygienists’ concrete, daily experiences. In this sense, Intact-Tooth becomes an innovative application, as it is the only one available today to perform an immediate assessment of the hard tissues of patients, providing protocols aimed at solving the problem and being consulted by the patient himself.

### Prevention and Treatment Protocols

As for prevention, it is advisable to reduce the intake of drinks and foods containing acid substances (fatty meats, fish and shellfish, meats and cold cuts, eggs, cheeses, coffee, tea, chocolate, fried foods, alcoholic beverages, beer and soft drinks), monitor the habit of smoking and alcohol, keep under control diseases such as gastroesophageal reflux and detect early eating disorders and psychiatric disorders, such as anemia and bulimia [5,6]. In addition, it is advisable to refrain from performing the daily methods of home oral hygiene after meals, as the lowering of pH caused by food creates an acid environment throughout the oral cavity: it takes about 30 min for the conditions of balance to be restored.

Once the type of patient has been identified, it is possible to formulate tailor-made clinical protocols: the practitioner will assess, at each appointment, the erosive state using the Intact-Tooth app, which will indicate the patient’s susceptibility and provide indications on the protocols and timing of the application of each product stated.

One of these involves using a carbonate-hydroxyapatite-zinc-substituted product (Biorepair Desensitizing Repair-Enamel Shock Treatment, Coswell S.p.A, Funo, Italy): patients are advised to apply it for 10 min a day, one week a month for three months [12].

It is also important to mention the problem found in patients undergoing orthodontic treatment, who are appropriately trained and motivated to the correct methods of home oral hygiene (electric/sonic toothbrush and aids for interproximal spaces such as dental floss/brush).

Patients are subsequently re-evaluated monthly for removing bacterial plaque with glycine/erythritol powders (non-cariogenic amino acid/sugar used in a mixture of air and water) and for removing any calcified deposits (calculus) using ultrasonic and manual instruments. If patients experience episodes of gum bleeding during the daily practice of oral hygiene, any sessions of ozone therapy (natural gas with high disinfectant power) and tubes of toothpaste with lactoferrin (antimicrobial protein) and hyaluronic acid, vitamin E (antioxidant properties) and *Hamamelis Virginiana* leaf extract (plant with astringent and antihemorrhagic properties) are recommended (Biorepair Plus Parodontgel); in addition, it may be helpful to ensure the daily intake of probiotics to promote the maintenance of a balanced microbiota (set of microorganisms present in the oral cavity) (Biorepair Peribioma) [13,14].

In orthodontic therapy, the risk of dental erosion is significantly increased. We are, therefore, recommended remineralizing toothpaste based on biomimetic nanohydroxyapatite (Biorepair Plus Total Protection or, in case of high sensitivity, Biorepair Plus Sensitive Teeth) or topical remineralizing products based on microrepair (Biorepair Desensitizing Repair-Enamel Shock Treatment) [12,15].

## 5. Conclusions

This first report on this application highlights its strengths, such as that it is the first to assess the health of the enamel, which allows professionals and patients to interact, improving patient compliance and providing effective and scientifically validated prevention and treatment protocols.

The future goals aim to refine more and more the doctor–patient relationship, thanks to the use of a clinical support device that can increase patient compliance, standardize home remineralization procedures and reduce the incidence of dental erosion and caries; the further goal, in favor of the clinician, is the gradual education of the learning machine to reach a self-diagnosis made by the application itself.

## Figures and Tables

**Table 1:** Shows the number of dentists, dental hygienists and undergraduate students who have used the application; RDH = registered dental hygienist; DDS = doctor of dental surgery.

User	*n*	%
RDH	1231	66.93855
DDS	463	25.17673
RDH Student	129	7.014682
DDS Student	16	0.870038
Total	1839	100

**Table 2:** Shows the number of patients who have used the application.

Patient	*n*	%
Male	1892	48.59
Female	2002	51.41
Total	3894	100

**Table 3:** Shows the number of patients in orthodontic therapy and not in orthodontic treatment.

Orthodontics	*n*	%
No treatment	3310	85
Multibracket treatment	584	15
Total	3894	100

**Table 4:** Shows the degree of susceptibility of the patients included in the application.

Susceptibility	Significance	*n*	%
0 to 2	No ETW	3522	90.45
3 to 8	Low	361	9.27
9 to 13	Medium	8	0.21
14+	High	3	0.08
Overall		3894	100

**Table 5:** Shows the statistical analysis results on age, BEWE index and degree of susceptibility.

	Mean	SD	Min	Median	Max
Age	36.72	14.52	6	35	36.72
BEWE	0.46	1.27	0	0	14
Grade	3.7	6.56	0	2	84

## Data Availability

Data are available upon reasonable request from the corresponding author.
